# Robotic disassembly of permanent magnet electric motors

**DOI:** 10.1098/rsos.241590

**Published:** 2025-03-19

**Authors:** Chaozhi Liang, D. T. Pham

**Affiliations:** ^1^ Department of Mechanical Engineering, School of Engineering, University of Birmingham, Edgbaston, Birmingham B15 2TT, UK

**Keywords:** robotic disassembly, permanent magnet motor, magnetomotive force, remanufacturing

## Abstract

This paper focuses on the disassembly of permanent magnet (PM) motors, which are the type of motor commonly used in electric vehicles (EVs). To handle the expected massive volume of PM motors available for remanufacturing as these EVs reach the end of their service life, efforts must be focussed on reducing manual labour during disassembly. In this study, the problem of removing a rotor from the stator in a PM motor using robots was explored. This is a challenging problem in PM motor disassembly because of the destabilising forces exerted by the magnets in the rotor. To prevent damage to the rotor and stator, an optimized disassembly path is generated using a model of the magnetic forces so that the rotor is centred relative to the stator while being pulled out. By following the optimized disassembly path and avoiding contact between the rotor and stator, the maximum disassembly force can be reduced by 49% and 38%, respectively, compared to when the rotor is off-centred or in contact with the stator.

## 1. Introduction

Remanufacturing is a process in which end-of-life (EOL) products are returned to at least their original condition, giving them a new lease of life while reducing raw materials, energy consumption and greenhouse gas emissions compared to manufacturing the same products anew [1]. The main processes in remanufacturing are product disassembly, component cleaning, component repair or replacement, product reassembly and product testing [2]. Disassembly is thus the first element in a remanufacturing process chain. It is labour-intensive as disassembly is difficult to automate. Where it can be successfully implemented, robotic disassembly improves work efficiency [3], reducing risks when dangerous products are involved.

The need to remanufacture EOL permanent magnet (PM) motors has increased in recent years owing to their availability and the desire to avoid sending them to landfills. PM motors are commonly used in electric vehicles, the quantity of which will reach more than 120 million in 2039 [4]. Remanufacturing PM motors enables the reuse of valuable components such as magnets made of rare earth materials.

The remanufacturing of EOL motors has been investigated for the UK market, and the lack of an automatic disassembling process has been highlighted [5]. In China, the remanufacturing of Y-series AC induction motors has been shown to reduce energy consumption by 68.26%, raw material usage by 75.32% and greenhouse gas emissions by 68.26% [6]. Related to disassembly techniques, a flexible vision-based method was developed to find and remove screws in electric motors automatically [7], and a novel image processing algorithm was proposed to enable a robot to detect screws and disassemble a motor [8]. A sequence planning software platform was developed for the robotic disassembly of electric motors [9] and the disassembly of a PM synchronous rotor was studied experimentally [10].

As far as the authors know, work on the robotic disassembly of electric motors has only been conducted empirically. There has been no systematic study of the disassembly process to determine the optimum disassembly strategy to minimize the efforts required and ensure the integrity of motor components. This paper reports on a fundamental investigation into a difficult task in PM motor disassembly, the separation of the rotor from the stator. Separating the rotor and stator is difficult because of the destabilizing forces exerted by the magnets in the rotor. The aim is to derive a model of the magnetic forces and generate an optimized disassembly path, ensuring that the rotor is centred relative to the stator and does not contact it while being pulled out.

The most common modelling methods in electromagnetism can be classified as analytical or numerical. Maxwell’s equations, Poisson’s equation, the magnetic scalar potential and the magnetic vector potential are the fundamental tools used to generate analytical models. Analytical modelling methods include equivalent circuit mapping, Schwartz Christoffel mapping, the harmonic method, the method of images and the surface charge and current model [11]. These analytical methods have been used to model magnetic field distributions in rotating magnet machines [12]. A motor model has been developed using the magnetomotive force (MMF) method in the framework of equivalent circuit mapping [13]. The main numerical tools employed in the study also include the finite element (FE) method and boundary element (BE) method. FE and BE are used to solve Laplace’s equation, which describes magnetic fields in space, and Poisson’s equation, which shows how they behave in different materials [14,15]. Analytical and numerical methods are well developed for modelling the magnetic force distribution for a rotor rotating inside a stator. However, there is a lack of models describing how the magnetic forces change when the rotor is pulled out of the stator during motor disassembly. Unlike the case of a rotating rotor, a huge change of resultant magnetic forces occurs in the process of removing the rotor from the stator.

The contributions of this work are: (i) developing a model to define the critical parameters affecting the robotic disassembly of PM motors; and (ii) generating a robotic automation solution for disassembling PM motors. Such an analysis has not been done so far, although it is critical to understanding and successfully implementing the robotic disassembly of PM motors. CCompared with the FE model, the MMF model requires less computation time and does not need powerful computing equipment to solve equations. Therefore, the MMF model can be integrated with robot control software to achieve fully automatic disassembly. In such an automated scheme, magnetic forces are measured during disassembly and are input to the MMF model. Optimum disassembly movements are then calculated using the model and sent to the robot for execution.

This paper is structured as follows. The problem of PM motor disassembly is described in §3. The MMF and FE methods are applied to model disassembly conditions in §4. Experiments to validate the models and the proposed disassembly solution are also described in §4. The results obtained are presented and discussed in §5. Section 6 concludes the paper.

## 2. Problem description

**Figure 1.**
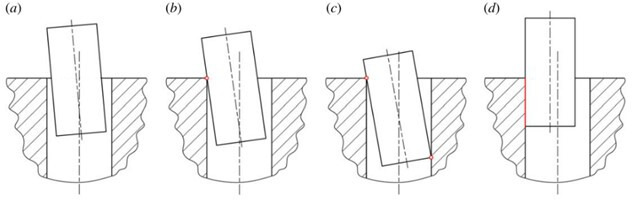
Peg-hole contact conditions: (*a*) no-contact, (*b*) one-point contact, (*c*) two-point contact, and (*d*) line contact [16].

The separation of the rotor and stator when disassembling PM motors can be simplified as the problem of removing a cylindrical peg from a cylindrical hole in the presence of a magnetic field. During disassembly, the peg may not be in contact with the hole, or it may touch the hole at one point or two points or along a line (figure 1) [16]. Disassembly can fail owing to jamming and wedging, which only occur under two-point contact conditions (contact state (*c*) in figure 1).

Jamming occurs when the peg cannot move owing to incorrectly applied forces and moments. Wedging involves the peg becoming stuck at a position because high internal forces within the peg keep it in static balance regardless of the external force. Wedging can happen only when the peg or the hole can deform elastically, and the clearance between the peg and the hole is sufficiently small relative to their diameters [17].

The ideal state during disassembly is having no contact between the peg and the hole, as there are no contact forces to cause them damage. In the case of a rotor and stator, this is difficult to achieve owing to the permeant magnetic forces that tend to cause the rotor to attach itself to the stator. Contact states (*b*–*d*) in figure 1 are, therefore, likely to occur, with contact state (*d*), the worst scenario, being the most probable.

In the ideal state of no contact, the rotor would levitate at the centre of the stator once both end bearings are removed. This would be possible if the magnetic fields at the poles were equal and the rotor were perfectly symmetrical. However, usually, the magnetic fields are unbalanced and the rotor geometry is imperfect. Therefore, without external intervention, the rotor will attach itself fully to the stator after the end bearings are removed, which explains the high likelihood of the line contact state (figure 1*d*).

Contact state (*d*) must be avoided because both the rotor and the stator can be damaged during disassembly. The strong magnetic attractive forces between the rotor and stator will generate high frictional resistance, causing excessive scoring and wear on the contact surfaces. This is also an issue with contact states (*b*) and (*c*). Scenario (*c*) is worse than (*b*) as it provides the necessary condition conditions for jamming and wedging, although. However, wedging is unlikely because the clearance between the rotor and stator is in the order of 0.7−1.5 mm, which is large for a 50−60 mm-diameter rotor.

This work aims to facilitate an ideal disassembly process where the rotor can be removed under contactless conditions to prevent damage. The next section describes the modelling of the forces acting on the rotor, the purpose of which is to direct a robot to pull the rotor away without the latter touching the stator.

## 3. Magnetic forces on the rotor during disassembly

### 3.1. Overall force analysis

**Figure 2.**
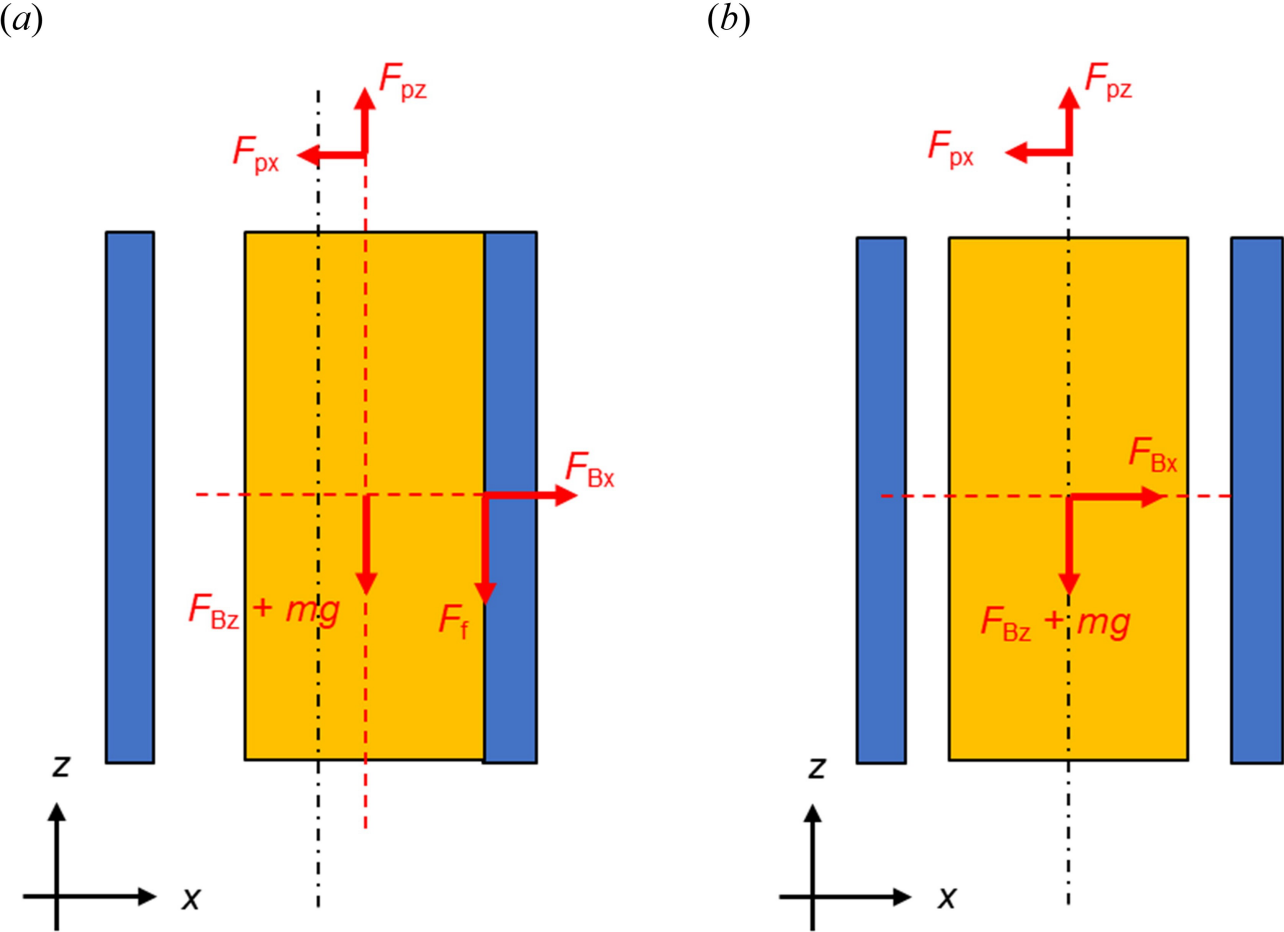
Force analysis underline: (*a*) line contact and (*b*) no-contact.

In the following analysis, for simplicity, slot structures are neglected, and the rotor and stator are assumed to be smooth cylinders. The rotor axis and stator axis are to be kept parallel throughout. Otherwise, contact state (*c*) will happen, incurring the risk of jamming. The disassembly force *F*_dis_ comprises two components, *F*_p*x*_ and *F*_p*z*_ (equation (3.1)). Under line contact conditions (figure 2*a*), *F*_p*x*_ and *F*_p*z*_ are given by equations (3.2) and (3.3), respectively, where *F*_B*x*_ and *F*_B*z*_ are the *x*- and *z*-components of the resultant magnetic force *F*_B_ on the rotor, mg is the weight of the rotor and *F*_f_ is the friction force on the rotor owing to *F*_B*x*_. *F*_f_ is given by equation (3.4), where $\mu_{f}$ denotes the dynamic coefficient of friction between the rotor and stator. The same equations apply under no-contact conditions, except that *F*_f_ is now zero (for symbol definitions, see table 2):


(3.1) $$F_{dis}=-\sqrt{F_{pz}^{2}+F_{px}^{2}},$$

(3.2) $${\;F}_{px}=-\left(F_{Bx}\right),$$

(3.3) $$F_{pz}=-\left(F_{Bz}+F_{f}+mg\right),$$

(3.4) $$F_{f}=\mu_{f}F_{Bx}.$$

The key to obtaining the total disassembly force is to determine the magnetic forces *F*_B_, which is the subject of the next section.

### 3.2. Determination of magnetic forces using the magnetomotive force method

The MMF method [13] was chosen over the magnetic scalar potential method [18] as it is simpler and gives more accurate results. This is because the latter method requires the solution of three-dimensional integral equations and produces large errors associated with divisions by small numbers, given the small distances involved (the gap between the rotor and the stator being less than a millimetre).

The MMF method was used first to model the magnetic force between a single bar magnet and a parallel steel bar. The result was then integrated to provide the force between a rotor comprising several bar magnets circumferentially positioned on a circular hub and a concentric steel cylinder representing the stator. It is assumed that the relative movement of the rotor and the hub is slow and magnetostatic conditions apply. Lenz’s law could, therefore, be neglected.

#### 3.2.1. Force between a bar magnet and a parallel steel bar

**Figure 3.**
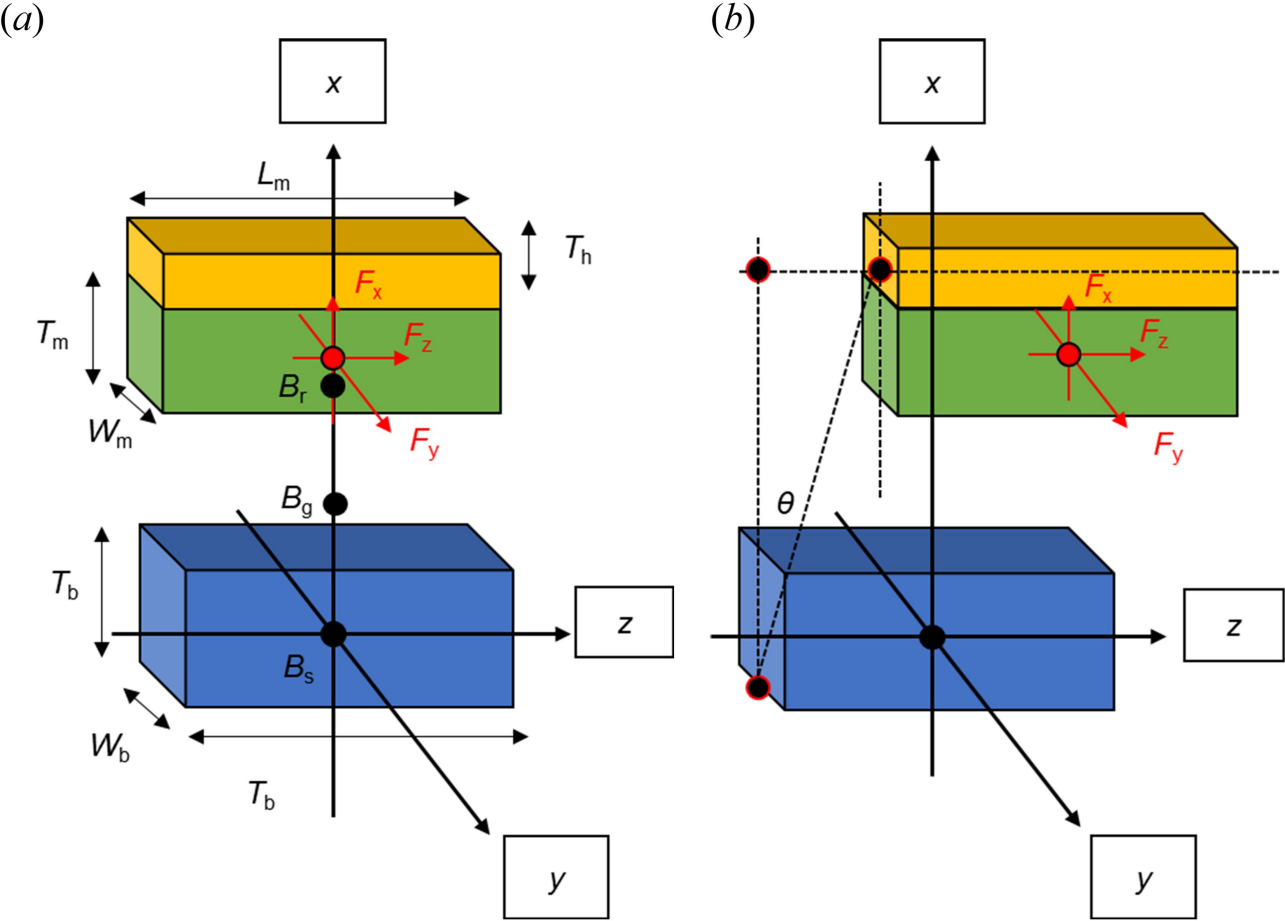
Single magnet and single steel bar problem: *(a)* magnet moves in *x* direction, and *(b)* magnet moves in *z* direction.


Both the magnet and the steel bar are modelled as made up of points. In the case of the magnet, the points are magnetic point sources. Referring to figure 3, the magnet (the green part) and its holder (the orange part) move along the *x* and *z* directions relative to the steel bar (the blue part). The magnetic flux diffuses from the point source ($B_{r}$) in the magnet equation (3.5) [13], through air (*$B_{g}$*) equation (3.6) [13], into the steel bar ($B_{s}$) equation (3.10), resulting in magnetic forces in the *x* direction (*$F_{x}$*) equation (3.11) and *z* direction ($F_{z}$) equation (3.12) on the steel bar. The magnetic flux ($B_{r}$) can be measured on the surface of the magnets using a Gauss meter:

(3.5) $$\left|M_{0}\right|=\frac{B_{r}}{\mu_{0}\mu_{r_{mag}}},$$

where $\mu_{0}$ is the magnetic permeability in vacuum, $\mu_{r_{mag}}$ is the relative magnetic permeability of the magnet and $M_{0}$ is the magnetization of the magnet. Both the magnetic north pole and south pole exert attractive forces on magnetic materials such as steel. Thus, the absolute value of magnetization is used to simplify calculations:


(3.6) $$B_{g}\left(x,z\right)=\frac{B_{r}}{d_{g}\left(z\right)+\frac{T_{m}}{\mu_{r_{mag}}}}\frac{T_{m}}{\mu_{r_{mag}}}\frac{A_{m}\left(x\right)}{A_{g}\left(x\right)},$$

where $B_{g}\left(x,z\right)$ is the magnetic flux density related to the position of the magnet in the *x* direction and *z* direction in the air gap. $A_{b}$$A_{m}$ represent the areas of the steel bar equation (3.7) and the magnet equation (3.8), respectively. $A_{m}$ is assumed to be the same as the area of the air gap $A_{g}$. These areas change depending on the position of the magnet in the *z* direction. $L_{b}$ and $L_{m}$ represent the length of the steel bar and the magnet, respectively. $W_{b}$ and $W_{m}$ represent the width of the steel bar and the magnet, respectively. $d_{g}\left(x\right)$ is the air gap size with the magnet located at *x*. $T_{m}$ is the thickness of the magnet:


(3.7) $$A_{b}\left(z\right)=\left(L_{b}-\left|z\right|\right)\times W_{b},$$

(3.8) $$A_{m}\left(z\right)=\left(L_{m}-\left|z\right|\right)\times W_{m},$$

(3.9) $$H_{g}\left(x,z\right)=\frac{B_{g}\left(x,z\right)}{\mu_{air}}=\frac{B_{g}\left(x,z\right)}{\mu_{0}\mu_{r_{air}}},$$

(3.10) $$B_{s}\left(x,z\right)=\text{0.2811}\times \text{log(}H_{g}\left(x,z\right)\text{)+ 0.2561}.$$

$H_{g}$ is the external magnetic strength in the air gap. $\mu_{air}=\mu_{0}\mu_{r_{air}}$ is the magnetic permeability of air. The magnetic hysteresis B–H curve is used to model how steel components react to magnetic fields. The steel bar used in the experiment was made of S235-grade carbon steel, but its B–H curve was unavailable to the authors. The B–H curve for 1018 low-carbon steel, a similar material, was used instead of equation (3.10). A correction factor ($\mu_{factor}$) was added to adjust the magnetic permeability of the material in the B–H region. $\mu_{factor}$ was determined by considering the difference between the magnetic force calculated using the MMF model and that experimentally measured.

The force *F*_B_ is given by equations (3.11) and (3.12):


(3.11) $$F_{Bx}\left(x,z\right)=-\frac{B_{g}{\left(z\right)}^{2}A_{b}\left(x\right)}{2\frac{B_{s}\left(x,z\right)}{H_{g}\left(x,z\right)\;\times \;\mu_{factor}}},$$

(3.12) $$F_{Bz}\left(x,z\right)=F_{Bx}\left(x,z\right)\times tan\;\theta \left(x,z\right),$$

where


(3.13) $$tan\;\theta \;\left(x,z\right)=\frac{z}{d_{g}\left(x\right)+T_{m}+T_{b}+\frac{T_{h}}{2}}.$$

$F_{Bx}$ and $F_{Bz}$ represent the resultant magnetic forces in the *x* and *z* directions, respectively. $T_{B}$, $T_{m}$ and $T_{h}$ are the thicknesses of the steel bar, the magnet and the magnet holder, respectively.

#### 3.2.2. Force between a bar magnet and a parallel steel bar

**Figure 4.**
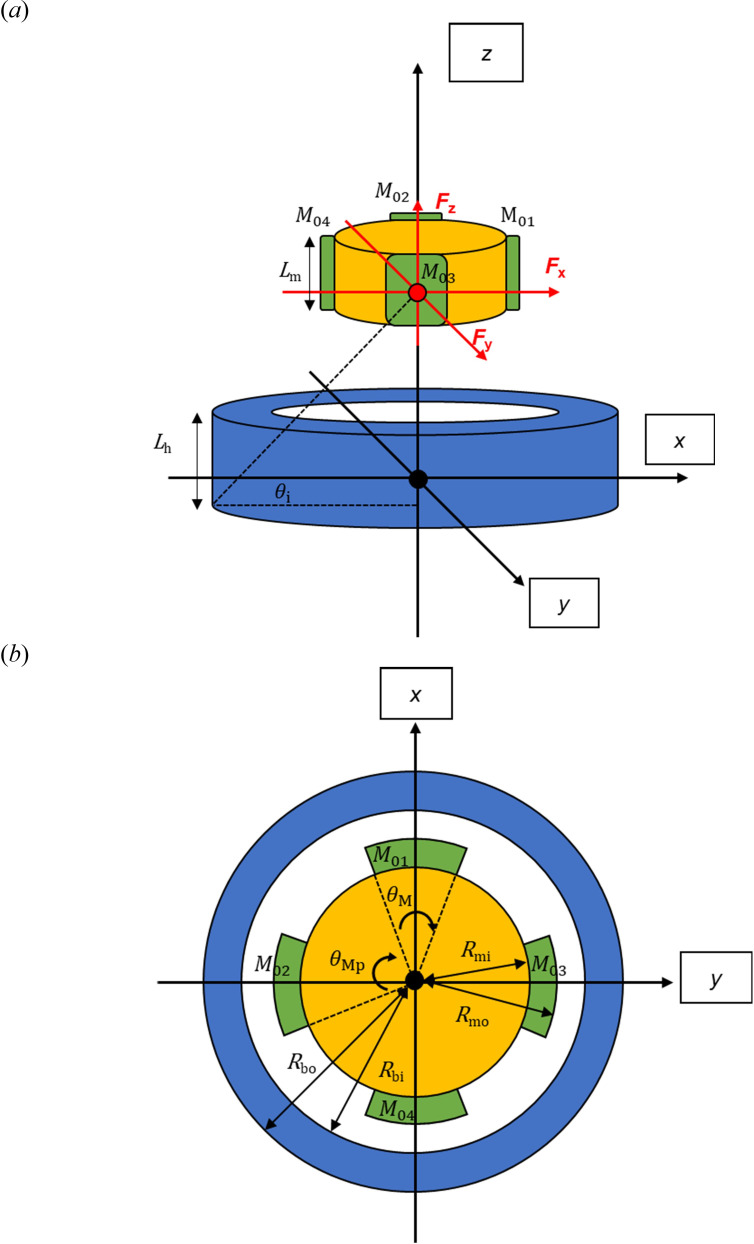
Cylindrical rotor and hub: (*a*) three-dimensional view, and (*b*) top view.

The above method was extended to the case where the rotor was made up of four bar magnets equidistantly positioned around a cylindrical holder as one common structure of a PM rotor [19]. This rotor assembly was able to move axially (along the *z* direction in figure 4*a*) and, to a limited degree, radially inside a cylindrical steel hub (along the *x* and *y* directions in figure 4*b*). The hub represents the stator. The equations describing the relative position between points on the magnets and corresponding points on the hub are given in appendix A.

Similar to the previous case, the resultant magnetic forces in the *x*, *y* and *z* directions ($F_{Bx}$, $F_{By}$ and $F_{Bz}$) can be calculated (equations (3.14)–(3.26)) [13].


(3.14) $$\left|M_{0}\left(i\right)\right|=\frac{B_{r}\left(i\right)}{\mu_{0}\mu_{r_{mag}}},$$

(3.15) $$B_{g}\left(i,x,y,z\right)=\frac{B_{r}\left(i\right)}{\left|d_{g}\left(i,x,y\right)\right|+\frac{T_{m}\left(i\right)}{\mu_{r_{mag}}}}\frac{T_{m}\left(i\right)}{\mu_{r_{mag}}}\frac{A_{m}\left(i,z\right)}{A_{g}\left(i,z\right)},$$

(3.16) $$A_{b}\left(i,z\right)=\left(L_{b}\left(i\right)-\left|z\right|\right)\times W_{b}\left(i\right)\times \frac{R_{bi}\times \pi \times \theta_{M}\left(i\right)}{180},$$

(3.17) $$A_{m}\left(i,z\right)=\left(L{\;}_{m}\left(i\right)-\left|z\right|\right)\times \;W_{m}(i)\times \frac{R_{mi}\times \pi \times \theta_{M}\left(i\right)}{180},$$

(3.18) $$H_{g}\left(i,x,y,z\right)=\frac{B_{g}\left(i,x,y,z\right)}{\mu_{air}}=\frac{B_{g}\left(i,x,y,z\right)}{\mu_{0}\mu_{r_{air}}},$$

(3.19) $$B_{s}\left(i,x,y,z\right)=\text{0.2811}\;\times \;log(H_{g}\left(i,x,y,z\right)\text{) + }\text{0.2561},$$

(3.20) $$F_{Bx}=F_{x1}-F_{x4},$$

(3.21) $$F_{By}=F_{y3}-F_{y2},$$

(3.22) $$F_{z}\left(i,x,y,z\right)=F_{x}\left(i\right)\times tan\;\theta \left(i,x,y,z\right)+F_{y}\left(i\right)\times tan\theta \left(i,x,y,z\right),$$

(3.23) $$tan\;\theta \left(i,x,y,z\right)=\frac{z}{d_{g}\left(i,x,y\right)+T_{m}\left(i\right)+T_{b}\left(i\right)+\frac{T_{h}}{2}},$$

(3.24) $$T_{m}\left(i\right)=R_{mo}\left(i\right)-R_{mi}\left(i\right),$$

(3.25) $$T_{b}\left(i\right)=R_{bo}\left(i\right)-R_{bi}\left(i\right),$$

(3.26) $$F_{Bz}=\sum_{i=1}^{i=4}F_{z}\left(i\right).$$

### 3.3. Determination of magnetic forces using the finite element method

**Figure 5.**
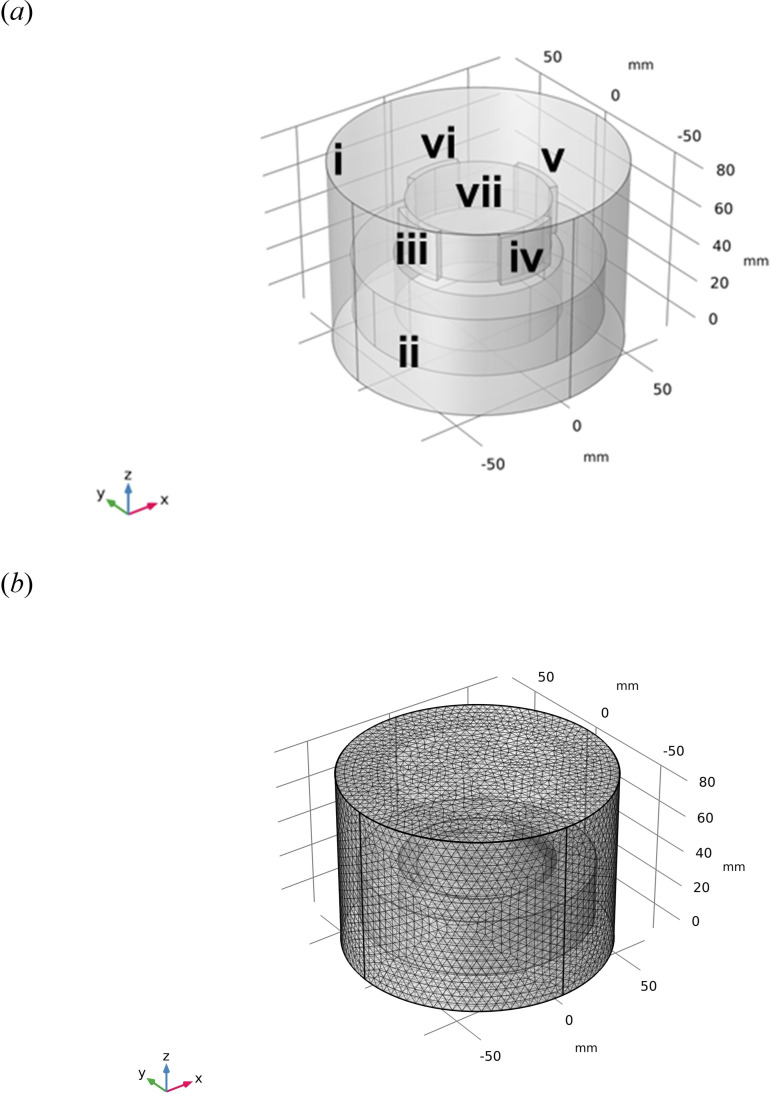
FE model of the cylindrical rotor–stator problem: (*a*) geometry: (i) air domain, (ii) steel hub, (iii) – (vi) magnets (*M*_01_, *M*_02_, *M*_03_ and *M*_04_) and (vii) magnet holder and (*b*) mesh


The problem of determining the forces on the magnetized rotor of figure 5 can be described by partial differential equations (PDEs). As the movement of the rotor is slow, the problem is a magnetostatics problem, and it is possible only to consider the magnetic forces in the FE model. The governing equations demonstrate how the magnetic flux transfers from the magnets (point sources), through the airgap, to the steel hub and produces magnetic forces on the hub [20]:

(3.27) ∇⋅B=0

(3.28) $$H=-\nabla \cdot V_{m},$$

(3.29) $$B_{1}n_{1}=B_{2}n_{2},$$

(3.30) $$B=\left\{ \begin{array}{l} \mu_{0}\left(H+M\right),in\;the\;magnet\\ \;\mu H,\;\;\;\;\;\;\;\;\;\;\;\;\;\;\;\;\;\;\;\;in\;the\;airgap\\ f\left(∥H∥\right)\frac{H}{∥H∥},\;\;in\;the\;steel\;hub \end{array}\right.,$$

(3.31) $$F=\oint_{\partial \Omega_{1}}^{,}n_{1}T_{2}dS,$$

(3.32) $$T_{2}=-pI-\left(\frac{\varepsilon_{0}}{2}E\cdot E+\frac{1}{2\mu_{0}}B\cdot B\right)I+\varepsilon_{0}EE^{T}+\frac{1}{\mu_{0}}BB^{T}.$$

Equations (3.27) and (3.28) are basic equations in magnetostatics, where **B** is the magnetic flux density; H is the external magnetic strength and $V_{m}$ is the magnetic scalar potential. The transfer of magnetic fields between different materials is described by equation (3.29), where n is the surface normal of the materials. Equation (3.30) gives the magnetization models of the magnet, air and steel hub, where **μ** is a matrix representing the magnetic permeability of the corresponding material. Equation (3.31) yields the magnetic force (F) on the steel hub, where $n_{1}$ is the surface normal of the hub and $T_{2}$ is the stress tensor of the surrounding air. Stress tensor of the surrounding air ($T_{2}$) is shown by equation (3.32), where p is air pressure, which is 0 in vacuum; I is the 3 by 3 identity matrix. E is the electric field, which is 0 in the current free model and $\varepsilon_{0}$ is permittivity in air.

The FE method implemented in the COMSOL Multiphysics 5.6 software was used to solve the above set of PDEs. The mesh sensitivity was tested to balance the accuracy of the results and the computational time. The following setting was adopted: a 0.5 mm triangular mesh was used on the surfaces of the magnets and the inner surface of the steel hub, and a triangular mesh in the range 0.209–4.87 mm controlled by the COMSOL software was employed for the rest of the components (figure 5*b*). The boundary of the air domain (i) in (figure 5*a*) was set to reduce calculation time. The magnetic flux density of each magnet was measured by a Gauss meter. As in the case of the analytical models, the B–H curve was also used to model how steel components react to a magnetic field. All the parameters (table 1) were built in COMSOL using a laptop with an Intel i7 10th generation processor, an RTX 2070MQ GPU and 32 GB of RAM.

**Table 1.** Parameters of the cylindrical rotor–stator problem.

no	parameters	symbol	values	unit
1	magnetization of magnet 1	$M_{01}$	−163.50	kA m^−1^
2	magnetization of magnet 2	$M_{02}$	162.24	kA m^−1^
3	magnetization of magnet 3	$M_{03}$	164.92	kA m^−1^
4	magnetization of magnet 4	$M_{04}$	−174.40	kA m^−1^
5	length of the steel hub	$L_{b}$	30	mm
6	length of the magnet holder	$L_{h}$	25	mm
7	length of the magnet	$L_{m}$	25	mm
8	inner radius of the steel hub	$R_{bi}$	39.5	mm
9	outer radius of the steel hub	$R_{bo}$	59.5	mm
10	radius of the magnet holder	$R_{h}$	33.5	mm
11	inner radius of the magnet	$R_{mi}$	33.5	mm
12	outer radius of the magnet	$R_{mo}$	37.5	mm
13	angle between magnets	$\theta_{Mp}$	90	degree
14	angle of cylindrical magnet	$\theta_{M}$	45	degree
15	weight of the magnets and the holder	m	0.327	kg
16	dynamic friction coefficient between the magnet and the hub	$\mu_{f}$	0.42	—
17	calculating factor for correcting the material magnetic permeability in the B-H region	$\mu_{factor}$	100	—
18	magnetic permeability of vacuum	$\mu_{0}$	4π10^-7^	H m^−1^

### 3.4. Experiments

**Figure 6.**
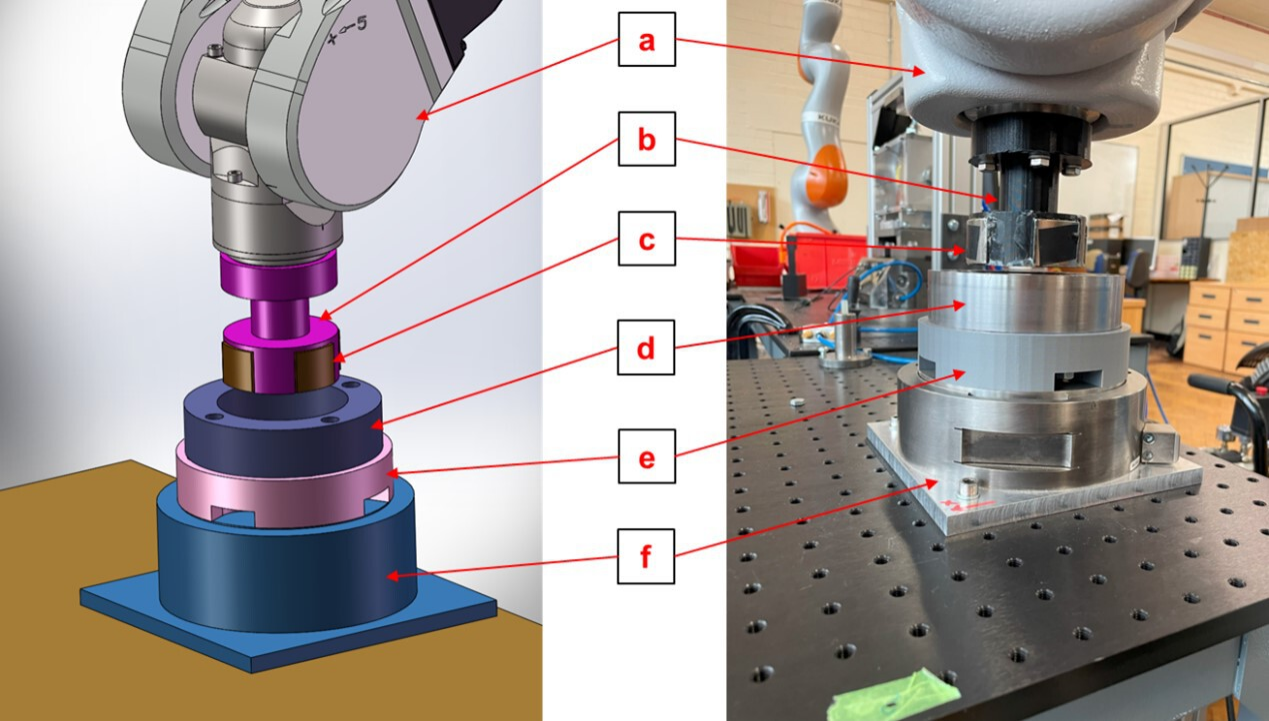
Experimental set-up for the cylindrical rotor–stator problem: *a,* Staubli TX90L Touch (robot); *b*, magnet holder; *c* , magnets; *d*, steel hub; e, holder of the steel hub and *f*, ATI Theta (FT sensor). Both the magnet holder and the holder of the steel hub were made of plastic, which did not affect the magnetic force.

Experiments were conducted to validate the MMF and FE methods as applied to the cylindrical rotor-stator problem. The experimental set-up is shown in figure 6. The magnet holder was made of three-dimensional-printed polylactic acid and the material of the steel hub was low-carbon steel. The movement of the rotor was controlled by a Staubli TX90L collaborative robot arm with a touch-sensitive cover, and the steel components were fixed on a table. Magnetic forces between the magnet and the steel hub were measured at different positions using an ATI force/torque (FT) sensor (model: Theta) also fixed on the table. The magnetic flux density of the magnets was measured by a Gauss meter (model: San Liang TS200). Static measurements were taken in the experiments; thus, Lenz’s law can be neglected. According to the manufacturer, the robot’s repeatability is 0.03 mm when the payload is 60 N. The maximum measured magnetic force was 14 N, and thus, it did not materially affect the accuracy of the experiment. Nevertheless, the robot was operated at 0.5% of its maximum speed to ensure its stiffness and precision.

To determine the centre position of the steel hub, the robot moved the rotor to touch several points on the hub’s outer perimeter. The external and internal surfaces of the hub were assumed to be concentric. Contact point coordinates were recorded when the FT sensor detected force changes just above 0.5 N. This process was repeated 10 times to obtain the positions of 10 different points on the steel hub on a plane normal to its axis. The coordinates of the recorded points were then used to locate the centre of the hub. Each experiment was repeated three times, and 300 data points were recorded for each rotor position. Table 1 lists all the parameters for the problem.

## 4. Results and discussion

**Figure 7.**
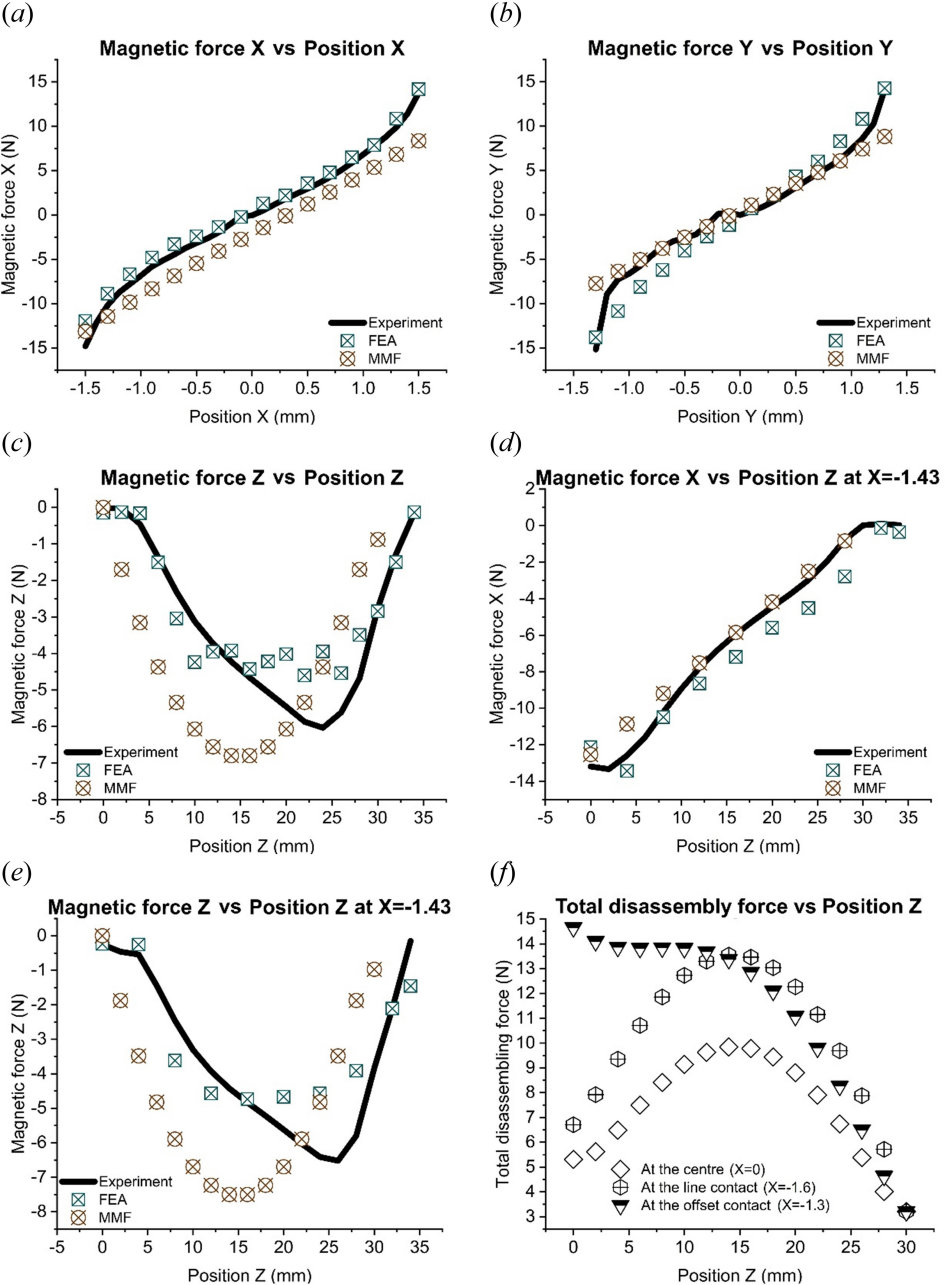
Validation of the experiment, the MMF and the FE methods and the results of the total disassembly force: (*a*) Magnetic force *x* vs Position *x*, (*b*) Magnetic force *y* vs Position *y*, (*c*) Magnetic force *z* vs Position *z*, (*d*) Magnetic force *x* vs Position *z* at *x* = -1.43 mm, (*e*) Magnetic force *z* vs Position *z* at *x* = -1.43 mm and (d) Total disassembly force.

Appendix B gives the FE simulation results for the cases of the rotor moving along the *x*, *y* and *x* directions relative to the stator. Figure 7 plots the magnetic forces and disassembly force at different rotor positions. The *x* movement range (−1.5 to 1.5 mm) and *y* movement range (−1.3 to 1.3 mm) are different because of the imperfect structure of the magnet holder in the experiment. The magnetic forces experienced in the positive directions (i.e. *x* = 0–1.5 mm; *y* = 0–1.3 mm) and negative directions (i.e. *x* = 0 to −1.5 mm; *y* = 0 to −1.3 mm) are caused by the different magnetic fields from the non-identical magnets (figure 7*a,b*). For movements in the *x*–*y* plane (i.e. the radial plane), with the rotor located inside the stator (at *z* = 0 mm), the forces obtained using the FE and MMF methods agree well with those measured experimentally (figure 7*a,b*). The FE method performed better when the rotor was moved along the *z* direction (i.e. the axial direction; figure 7*c*). However, it is difficult to determine the magnetic forces accurately using the MMF method when the magnet is located near the top of the steel hub (*z* ≈ 30 mm), where the magnetic flux concentrates at the corner of the magnet (figure 7*c,e*). Figure 10*f* in appendix B2 shows that the FE method can handle this corner effect. From figure 7, it can also be seen that the peak magnetic forces obtained by the two methods are similar.

**Figure 9.**
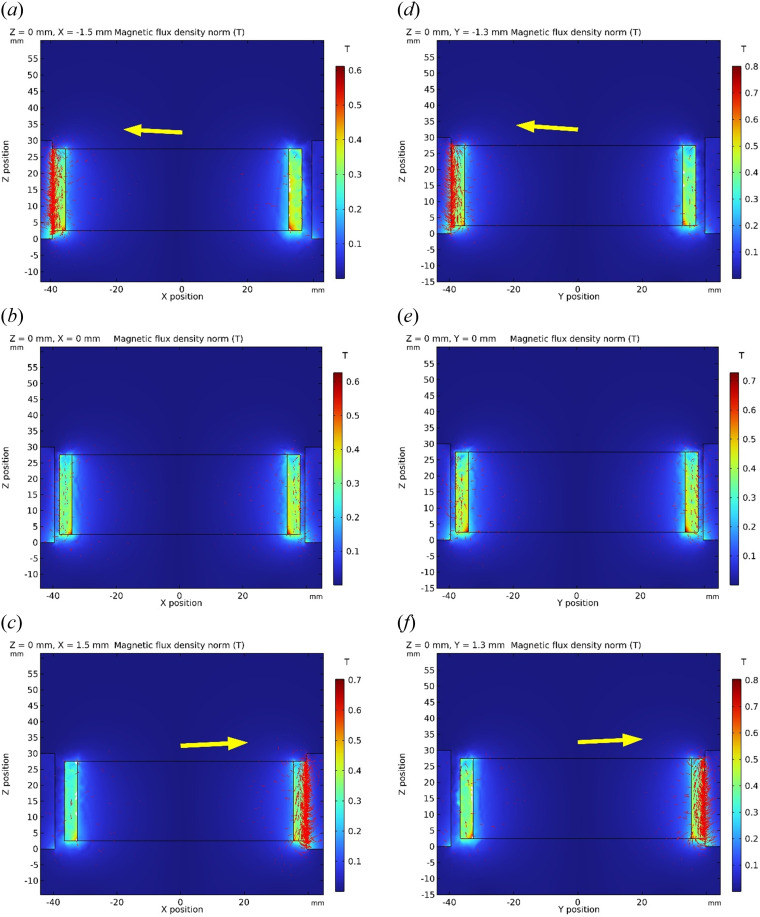
Rotor motion in the *x* and *y* directions: (*a*) at *z* = 0 mm; *x* = - 1.5 mm; *y* = 0 mm, (*b*) at *z* = 0 mm; *x* = 0 mm; *y* = 0 mm, (*c*) at *z* = 0 mm; *x* = 1.5 mm; *y* = 0 mm, (*d*) at *z* = 0 mm; *x* = 0 mm; *y* = - 1.3 mm, (*e*) at *z* = 0 mm; *x* = 0 mm; *y* = 0 mm and (*f*) at *z* = 0 mm; *x* = 0 mm; *y* = 1.3 mm. The colour table represents the magnetic flux density norm. The red arrows represent the proportional magnetic flux density in the *x*, *y* and *z* directions, and the yellow arrow represents the proportional resultant magnetic force of the magnet.

**Figure 10.**
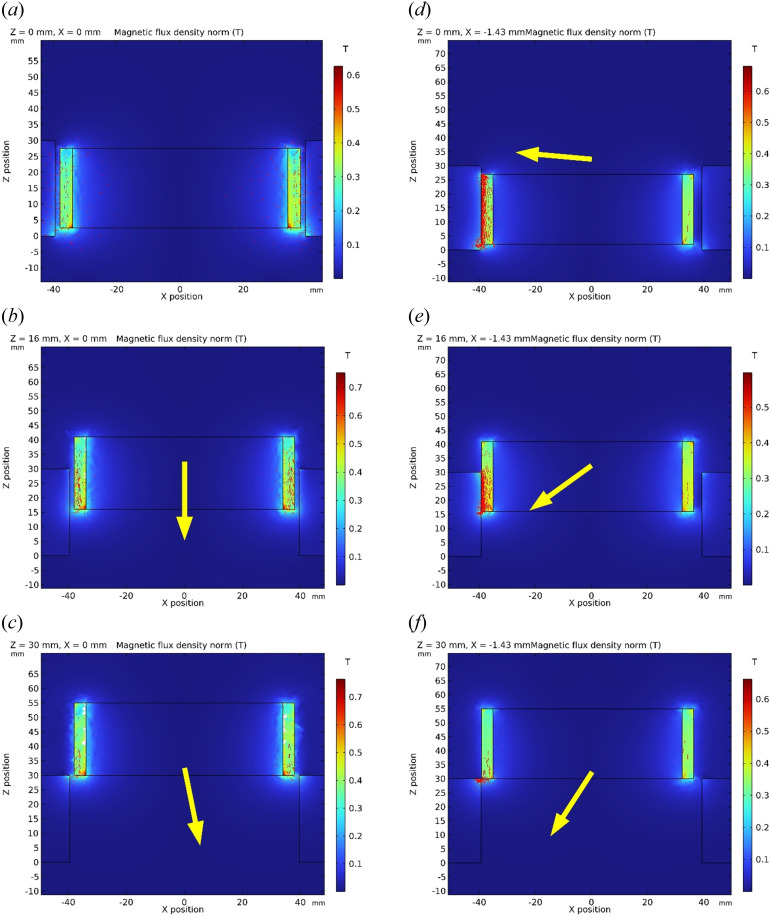
Rotor motion in the *z* direction: (*a*) at *z* = 0 mm; *x* = 0 mm; *y* = 0 mm, (*b*) at *z* = 16 mm; *x* = 0 mm; *y* = 0 mm, (*c*) at *z* = 30 mm; *x* = 0 mm; *y* = 0 mm, (*d*) at *z* = 0 mm; *x* = 1.43 mm; *y* = 0 mm, (*e*) at *z* = 16 mm; *x* = -1.43 mm; *y* = 0 mm and (*f*) at *z* = 30 mm; *x* = -1.43 mm; *y* = 0 mm. (*a*), (*b*) and (*c*) with the rotor and stator remaining concentric, and (*d*), (*e*) and (*f*) with the rotor axis offset by −1.43 mm along the *x* direction relative to the stator axis. The colour table represents the magnetic flux density norm. The red arrows represent the proportional magnetic flux density in the *x*, *y* and *z* directions, and the yellow arrow represents the proportional resultant magnetic force of the magnet*.*

To find the optimal disassembly path, the magnetic forces on the rotor are considered. The resultant magnetic forces along *x* or *y* are significantly increased when the magnets are close to the left or the right side of the hub as the magnetic fields concentrate on one side (figure 9*a,c,d,f*). However, the forces are smaller at the centre of the steel hub because, there, the magnetic forces cancel out (figure 9*b,e*). Moreover, radial movements in the *x* and *y* directions cause significant changes in the magnetic force when comparing the offset position (*x* = −1.43) and central position (figure 7*a,b*). However, they have limited effects on the axial magnetic force *z* (figures 7*c,e*).

The total disassembly force is minimal at the centre of the steel hub (9.84 N at *x* = 0 mm) compared to that in the contact condition (13.14 N at *x* = −1.6 mm) and the offset condition (14.33 N at *x* = −1.3 mm) figure 7*f*. Thus, compared with the disassembly force under the contact condition and the offset condition, the maximum disassembly force in the balanced non-contact case is, respectively, 38% and 49% smaller. The best way for a robot to remove the rotor would be to lift it out of the steel hub while keeping it concentric with the hub (i.e. *x* = *y* = 0 mm). In addition to requiring the minimum disassembly force, this also prevents surface damage to both components. However, note that this experiment used a three-dimensional-printed rotor, new magnets and a CNC (computer-numerical-control)-precision-machined steel hub. The structure was, therefore, almost perfectly symmetrical, and the magnetic fields were similar. However, the conditions of EOL electric motors are poorer, and the magnetic fields are dissimilar. Thus, in practice, it will be necessary to search for the optimal position for disassembly.

## 5. Conclusion and future work

In conclusion, a model to calculate the magnetic forces acting on a rotor in a PM motor has been generated using the MMF method. Compared with the FE method, the MMF method is a fast model for computing magnetic forces. However, it is difficult for the MMF method to accurately determine magnetic forces along the axial direction of the rotor owing to corner effects. The FE method enables corner effects to be accounted for but requires high-performance computers and a long computational time.

Because of its efficiency, the developed MMF model is suitable for real-time use to guide a robot in disassembling a PM motor. In such a robotic disassembly scenario, forces measured during disassembly are input to the MMF model to calculate optimal robot movements to minimize damage. Note that, this control method is appropriate for applications where proprietary industrial robots are employed, which typically only accept position control information from users.

The optimal disassembly strategy is to keep the rotor concentric with the stator to eliminate radial magnetic forces on the rotor while withdrawing it axially. Theoretically, in this central position, the magnetic force along the radial direction vanishes, so the rotor does not attach to the stator, and the total disassembly force consists only of the weight of the rotor and the magnetic force along the axial direction. Thus, the maximum disassembly force is lower than the disassembly forces under the contact and offset conditions. Therefore, this strategy helps reduce the total disassembly force and avoids damage to the rotor and stator surfaces.

However, in practice, challenges remain owing to the unstable magnetic fields and the imperfect symmetry of the rotor and stator structures. Thus, the rotor position where lateral magnetic forces are minimized might not be at the exact centre of the stator. To take these practical imperfections into account, future work might focus on improving the accuracy of the MMF method by experimentally determining the magnetic permeability of the steel hub.

## Figures and Tables

**Figure 8.**
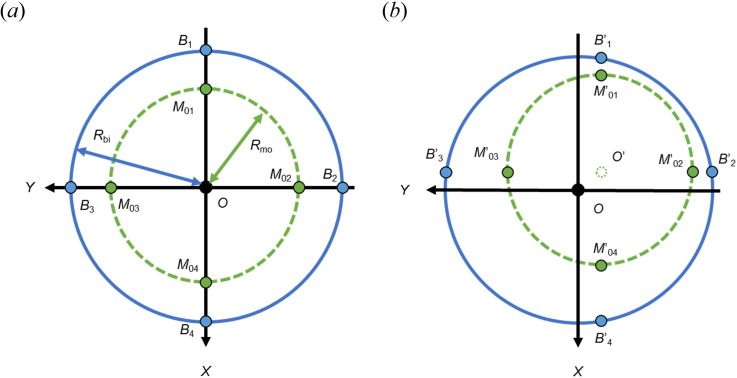
Relative position between points on the magnets and corresponding points on the hub: (*a*) two-dimensional view (concentric position) and (*d*) two-dimensional view (offset position).

## Data Availability

Please find a MATLAB code file for the MMF method and results for figure 7 on this link: [21].

## Appendix A

Each magnet has an aligned point on the steel hub on the top view (on the *xy* plane; figure 8) and the positions of the steel hub change when the positions of the magnets change (figure 8*b*). The positions of the four magnets are projected to a global Cartesian coordinate system equation (A 1). The magnets are allowed to move in the *x* or *y* direction in the hub, creating a new coordinate system ($O^{\prime }$) equation (A 6). The magnet and steel points are assumed to be always aligned because misalignments result in a reduction in magnetic forces in this model. The steel point is calculated based on the new coordinates of the magnets. Therefore, linear equations are created to calculate the coordinates of the steel points ($P_{B}^{\prime }$) by using the coordinates of the magnets ($P_{M}^{\prime }$), the inner radius of the hub ($R_{bi}$ and the new origin point ($O^{\prime }$) (equations (A 7)–(A 9)). The air gaps (g) are calculated by using the coordinates of the steel points ($P_{B}^{\prime }$) and the coordinates of the magnets ($P_{M}^{\prime }$) equation (A 10):

(A 1)
$$P_{M}^{\prime }\left(i\right)=\left(x_{M}^{\prime }\left(i\right)sin\theta_{Mp}\left(i\right),y_{M}^{\prime }\left(i\right)cos\theta_{Mp}\left(i\right)\right),$$

where the coordinate position of each magnet is:

(A 2)
$$P_{M_{1}}^{\prime }=\left(x_{0}+x-R_{mi},y_{0}+y\right),$$

(A 3)
$$P_{M_{4}}^{\prime }=\left(x_{0}+x+R_{mi},y_{0}+y\right),$$

(A 4)
$$P_{M_{2}}^{\prime }=\left(x_{0}+x,y_{0}+y-R_{mi}\right),$$

(A 5)
$$P_{M_{3}}^{\prime }=\left(x_{0}+x,y_{0}+y+R_{mi}\right),$$

$x_{M}$ and $y_{M}$ are the coordinates of the magnets. $x_{0}$ and $y_{0}$ are 0, representing the origin point of the global coordinate system. *x* and y are movements of the rotor in the *x* or *y* directions, respectively. $R_{mo}$ is the outer radius of the magnet:

(A 6)
$$O^{\prime }=\left(x_{0}^{\prime },y_{0}^{\prime }\right)=\left(x_{0}+x,y_{0}+y\right),$$

(A 7)
$$P_{B}^{\prime }(i)=\sqrt{{\left(x_{B}(i)-x_{0}^{\prime }\right)}^{2}+{\left(y^{\prime }(i)-y_{0}^{\prime }\right)}^{2}}=R_{bi},$$

(A 8)
$$y_{B}^{\prime }\left(i\right)=\frac{x_{B}^{\prime }\left(i\right)-x_{M}^{\prime }\left(i\right)}{O_{x}^{\prime }}\times \left(O_{y}^{\prime }-y_{M}^{\prime }\left(i\right)\right)+y_{M}^{\prime }\left(i\right),$$

(A9)
$$x_{B}^{\prime }(i)-x_{0}^{\prime }=\sqrt{R_{bi}{,}^{2}-{\left(y^{\prime }{,}_{B}(i)-y_{0}^{\prime }\right)}^{2}},$$

(A 10)
$$d_{g}\left(i,x,y\right)=\sqrt{{\left(x_{B}^{\prime }(i)-x_{M}^{\prime }\left(i\right)\right)}^{2}+{\left(y_{B}^{\prime }(i)-y_{M}^{\prime }\left(i\right)\right)}^{2}}.$$

where $O^{\prime }$ is the new origin point. $x_{M}^{\prime }$ and $x_{B}^{\prime }$ are the new *x* coordinates of the magnet or steel point, respectively, and $y_{M}^{\prime }$ and $y_{B}^{\prime }$ are the new *y* coordinates of the magnet or steel point, respectively.

## Appendix B

### Appendix B.1.

Cylindrical rotor–stator problem—rotor moving in the *x* and *y* directions; figure 9.

### Appendix B.2.

Cylindrical rotor–stator problem—rotor moving in the *z* direction with the rotor and stator remaining concentric and with the rotor axis offset by −1.43 mm along the *x* direction relative to the stator axis; figure 10.

## Symbols

See table 2.

**Table 2.** Symbols and examples are listed as follows.

$A_{b}$	surface area of the steel bar (m^2^)
$A_{g}$	surface area of the air gap (m^2^)
$A_{m}$	surface area of the magnet (m^2^)
$A_{s}$	surface area of the steel (m^2^)
B	magnetic flux density of materials in the FE model (T)
$B_{g}$	magnetic flux density in the air gap (T)
$B_{r}$	magnetic flux density at the magnet (T)
$B_{s}$	magnetic flux density at the steel (T)
$d_{g}$	length of the air gap (m)
E	electric field in the FE model (V/m)
F	magnetic force in the FE model (N)
$F_{dis}$	total disassembly force (N)
$F_{f}$	frictional force (N)
$F_{px}$	unplugging force in the *x* direction, (N)
$F_{pz}$	unplugging force in the *z* direction, (N)
$F_{x}$	magnetic force along the *x* direction, (N)
$F_{y}$	magnetic force along the *y* direction, (N)
$F_{z}$	magnetic force along the *z* direction, (N)
$F_{Bx}$	resultant magnetic force on the rotor along the *x* direction, (N)
$F_{By}$	resultant magnetic force on the rotor along the *y* direction, (N)
$F_{Bz}$	resultant magnetic force on the rotor along the *z* direction, (N)
g	gravitational constant, 9.81 (m s^−2^)
H	magnetic strength of material in the FE model (kA m^−1^)
$H_{g}$	magnetic strength at the air gap (kA m^−1^)
$H_{s}$	magnetic strength at the steel bar (kA m^−1^)
I	the identity 3-by-3 tensor (or matrix) in the FE model
i	number of magnets
$k_{cal}$	calculating factor for the magnetic forces
$L_{b}$	length of the steel bar (m)
$L_{h}$	length of the magnet holder (m)
$L_{m}$	length of the magnet (m)
n	surface normal of material in the FE model
M	magnetic intensity of the magnet in the FE model (kA m^−1^)
$M_{0}$	magnetic intensity of the magnet (kA m^−1^)
m	weight of the rotor (kg)
$O^{\prime }$	the origin after movement (m)
$O_{x}^{\prime }$	new *x* coordinate of the origin after movement (m)
$O_{y}^{\prime }$	new *y* coordinate of the origin after movement (m)
$P_{B}^{\prime }$	new coordinate of the steel hub after movement (m)
$P_{M}^{\prime }$	new coordinate of the rotor after movement (m)
p	air pressure in the FE model (Pa)
$R_{bi}$	inner radius of the steel hub (m)
$R_{bo}$	outer radius of the steel hub (m)
$R_{mi}$	inner radius of the magnet (m)
$R_{mo}$	outer radius of the magnet (m)
T	stress tensor of material in the FE model
$T_{b}$	thickness of the magnet (m)
$T_{h}$	thickness of the magnet holder (m)
$T_{m}$	thickness of the magnet (m)
$V_{m}$	magnetic scalar potential in the FE model (A)
x	position in the *x* direction (m)
$x_{0}$	*x* coordinate of the original origin (m)
$x_{0}^{\prime }$	*x* coordinate of the new origin after movement (m)
$x_{B}^{\prime }$	*x* coordinate of the steel hub after movement (m)
$x_{M}^{\prime }$	*x* coordinate of the magnet after movement (m)
$x_{g}$	air gap (m)
y	position in the *y* direction (m)
$y_{0}$	initial *y* coordinate of the origin
$y_{0}^{\prime }$	*y* coordinate of the new origin after movement (m)
$y_{B}^{\prime }$	*y* coordinate of the steel hub after movement (m)
$y_{M}^{\prime }$	*y* coordinate of the magnet after movement, (m)
$W_{b}$	width of the steel bar (m)
$W_{h}$	width of the magnet holder (m)
$W_{m}$	width of the magnet (m)
z	position in the *z* direction (m)
$\varepsilon_{0}$	permittivity in air in the FE model (F m^−1^)
μ	permeability of the corresponding material in the FE model (H m^−1^)
$\mu_{0}$	permeability of vacuum (H m^−1^)
$\mu_{f}$	dynamic frictional coefficient
$\mu_{factor}$	calculating factor for correcting the material magnetic permeability in the B–H region.
$\mu_{air}$	permeability of air (H m^−1^);
$\mu_{steel}$	permeability of steel (H m^−1^);
$\mu_{r_{mag}}$	relative permeability of the magnet;
$\mu_{rair}$	relative permeability of air;
$\mu_{rsteel}$	relative permeability of carbon steel;
θ	angle between the centre of the magnet and the centre of the steel bar (°)
$\theta_{M}$	angle of the cylindrical magnet (°)
$\theta_{Mp}$	angle between magnets (°)
